# Recognizing and appraising symptoms of breast cancer as a reason for delayed presentation in Ghanaian women: A qualitative study

**DOI:** 10.1371/journal.pone.0208773

**Published:** 2019-01-09

**Authors:** Adwoa Bemah Bonsu, Busisiwe Purity Ncama

**Affiliations:** 1 Discipline of Nursing, School of Nursing and Public Health, College of Health Sciences, University of KwaZulu-Natal, Durban, South Africa; 2 Department of Nursing, Faculty of Allied Health Sciences, College of Health Sciences, Kwame Nkrumah University of Science and Technology, Kumasi, Ghana; Weill Cornell Medical College in Qatar, QATAR

## Abstract

**Background:**

The burden of late presentation is well established in women presenting with advanced breast cancer in Africa. This paper aims to explore the reasons for delayed presentation in Ghanaian women with breast cancer.

**Method:**

Eleven (11) women diagnosed with advanced breast cancer were purposively sampled within three years of diagnosis at the palliative care clinic of the Komfo Anokye Teaching Hospital, Ghana. Participation was voluntary. Data was collected through in-depth interviews using a self-devised semi-structured interview guide. The interviews were conducted in “Twi” (local language), audio-tape recorded and covered the women’s journey from symptom discovery to their intention to seek help. All audio-taped interviews were transcribed based on the meaning of the respondents’ comments. The data was managed using Nvivo version 11 qualitative software. Data was analyzed concurrently with data collection applying the principles of thematic analysis.

**Key findings:**

All the women delayed presentation due to overlapping reasons. Symptom appraisal among the women occurred in two main stages: individual understanding of breast symptom and interactive understanding of the breast symptom. These stages were based on cognitive, psycho-cultural and social factors. The five main themes generated from the data were: symptom experience, knowledge of breast cancer, role of social life and network, coping with a breast symptom and lastly intent to seeking health care. A conceptual model was developed to illustrate the relationships among the key factors and concepts emanated from this study.

**Conclusion:**

Recognition and appraisal of breast cancer symptom in the eleven (11) Ghanaian women interviewed in this study was poor. For instance, a painless breast lump was considered not serious until a sensory symptom appears. This led women to experience appraisal and time point intervals. To minimize the incidence of late presentation of breast cancer cases in Ghana, adequate educational intervention should be provided for Ghanaian women and their social network, and other stakeholders.

## Introduction

Globally, breast cancer (BC) is the top cancer among women and is increasing in developing countries [1]. The burden with late diagnosis is well established in women presenting with advanced breast cancer (ABC) in African countries such as Ghana [2, 3]. Breast cancer is the most common cancer in Ghanaian women and the leading cause of cancer related deaths [4]. Ghana lacks a population-based cancer registry [5]; however, available data confirm that about 50–80% Ghanaian women with the disease are diagnosed late with ABC and have poor survival outcomes [2, 6, 7].

Due to infrastructural challenge and cost, mammography screening is not practical and feasible in Ghana [8, 9]. In essence, higher proportion of BC cases are seen in self-discovered symptomatic women [9, 10], independent of a structured breast self-exam [11]. Thus, achieving early diagnosis of BC in Ghana largely depends on well-informed women who self-refer as well as clinicians that practice opportunistic screening [9, 11, 12]. However, Ghanaian women wait for about 8 to 14 months before care seeking for a discovery of a breast cancer symptom [7, 9].

Data has shown a significant correlation between late presentation and survival [13] and this has influenced rigorous scientific approaches to identify possible facilitators of time interval that could potentially influence the development of interventions to help reduce late presentation [14–17]. Yet, not much has been done to facilitate the development of effective policies on this phenomenon [14, 18], especially, within the socio-cultural context of Ghana [19].

Two frameworks posited to describe symptom appraisal and health seeking are the Andersen model of total patient delay [20] and the model of pathways to treatment [21]. Indeed, the latter is more recent and acknowledges existing psychological theory such as the Common Sense Model of illness Self-regulatory within the context of appraisal and health-seeking intervals [21]. However, the Andersen model uniquely highlights the role of appraisal or misattribution of a symptom as a significant determinate of delay in help seeking. The authors defined delay in a broader context that transcend beyond the patient, referred as total patient delay [20]. Total patient delay has been classified into five (5) stages to include appraisal delay, illness delay, behavioral delay, scheduling delay and treatment delay [20]. Appraisal delay begins with the recognition of bodily symptoms to tagging it as a serious symptom. In the definition of delay, health provider (system) delays were also noted as significant [20, 22]. However, appraisal delay is described as the most significant phase accounting for 60% of patient total delay [12, 20]. The principal tenets of this study is in consonance with Andersen’s model of total patient delay which underscores the role of appraisement and misattribution of a BC symptom as a key determinant of appraisal interval in seeking help within the socio-cultural context of Ghana [20]. In essence, the Andersen model was adopted as the conceptual framework for this study. The model seems more appropriate to extensively explore the Ghanaian woman’s experience with symptom recognition and appraisal as well as the potential factors impeding prompt decision to seek care and early detection of BC.

Care-seeking decisions among women have been noted to be influenced by women’s own experiences with prior breast concerns, cognitive, psychological and socio-cultural factors [14, 23]. Data suggests that appraisal interval beyond three (3) months adversely affects BC survival [13]. Potential facilitators for appraisal and time intervals commonly cited in the literature include but not limited to poor knowledge on BC, healthcare inaccessibility, and maladaptive coping [14, 17, 18] However, studies on this phenomenon from African communities such as Ghana, where BC incidence seems rising with more women presenting with advanced disease, resources are limited, and structured screening services are uncommon or appear scanty [14, 23]. Evidently, Ghanaian women’s perspective on this phenomenon is missing in the literature. This study therefore explored the experiences of Ghanaian women diagnosed with advanced breast cancer (ABC) within the context of symptom recognition and appraisal. To the best of our knowledge, this study seems the first to employ a qualitative approach to hear the stories of Ghanaian women diagnosed with ABC in a tertiary institution well-equipped with BC treatment facilities. This study affords a unique opportunity to tailor future studies and BC interventions in Ghana.

## Materials and methods

### Design

The study used a qualitative exploratory descriptive approach to answer the research question: what are the experiences of women in recognizing and appraising symptoms of BC as a reason for delayed presentation? Employing this design, the symptom appraisal of women diagnosed with ABC was explored, understood and described [24]. This design was useful since little is known about the phenomenon in Ghana [25].

### Setting

The study was conducted at the Komfo Anokye Teaching Hospital (KATH), Kumasi. This is one of the largest teaching hospitals located in the northern sector of Ghana, serving patients across the country and has a bed capacity of about 1200. It has 12 clinical directorates with several units including the palliative care unit of the Family Medicine Directorate which served as the recruitment outlet.

### Population and sampling technique

Women diagnosed with ABC referred to the palliative care clinic of KATH constituted the study population. About 120 patients diagnosed with terminal conditions are referred to the palliative clinic yearly (KATH Palliative Clinic Record, 2017). Of these, eleven (11) women diagnosed with ABC and refereed to the palliative clinic within three (3) years were purposively sampled. Purposive sampling ensured that women diagnosed with either stage III or IV BC were recruited into the study to share their living experiences on the phenomenon of interest [25]. Further, women who were not too ill to communicate and those who could speak in either “Twi” (local language) or English (languages the lead author understands and could speak fluently) were enrolled. To reduce recall bias, women recruited for the study were diagnosed at different periods. Written consent was obtained from all the women. All women who officially agreed to be included completed the study, and saturation of data was achieved with eleven (11)) women, with no new themes emerging [26].

### The interview

Data collection started from January, 2018 through to June, 2018, with a face–to-face in-depth interview with each respondent using a semi-structured interview guide [25]. The guide centered on the journey of the women from discovery of BC symptoms to inferring as ill. Also, experiences on the decision to seek cancer care were explored. Responses were probed further or where necessary, re-directed to ensure complete understanding of the respondents’ accounts and also to make sure that interviewees responded in accordance with the study objective [26]. All interviews were conducted in ‘Twi’, the language most comfortable for the respondents and transcribed based on the meaning of the respondents’ comments [27]. To ensure the accuracy of translation, the transcription was discussed with a person competent in “Twi” and English languages while maintaining confidentiality. Date, time and venue of interviews were scheduled to suit respondents. All the participants were living with ABC and depended on their families for physical, emotional, financial and psycho-social support. They lived with their families in self-contained apartments and preferred to stay indoors to avoid being seen in their frail state and attract any unfavorable comments from the public. Hence, they visited the hospital for supportive care services only on appointment dates. In view of this, most of the participants (9) preferred to be interviewed at the comfort and privacy of their homes without any intrusion of family members. The interviews lasted for 60 to 90 minutes and were audio-tape recorded with respondents’ approval. Field notes were taken during each interview to include non-verbal signs, researcher’s reflections and main concerns of respondents [28]. The interview guide was pre-tested with 3 women diagnosed with ABC receiving care at the Oncology clinic at KATH. This helped us to ensure the appropriateness of the guiding questions. The interviews were conducted by the first author (ABB), a qualitative researcher with clinical and academic experience in oncology practice. As a researcher, ABB speaks and writes both ‘Twi’ and English languages. The interviewer does not work at the palliative care clinic; hence she had no direct influence on the study setting and the participants.

### Data analysis

Employing the processes of thematic analysis [28, 29], data analysis was done concurrently with data collection. This helped to achieve data saturation. Transcripts were read repeatedly to make meaning of the respondents’ realities and NVivo version 11, a qualitative software was used to manage the data. Initial emerging themes were followed up in subsequent interviews and supported with field notes to fully develop themes. To check the accuracy of the transcription, member check was done with four respondents [29]. The lead author (ABB) initially analyzed the data, and the second author (BPN) confirmed the findings to ensure that the respondents’ realities were accurately and truly represented, with discourse discussed.

### Trustworthiness/Rigor

Credibility, dependability, confirmability and authenticity were used to ensure the trustworthiness of the study [30]. Member checks with four (4) respondents coupled with concurrent data analysis ensured the full understanding and accurate presentation of respondents’ realities. Replication of the study and potential applicability of the findings in similar setting was achieved by detailed description of the study setting, design, methodology as well as the respondents’ background. Further, taking detailed field notes at each interview and discussion of study findings among authors ensured auditability of the study [31].

### Ethics

Ethical approval was sought from the Biomedical Research Ethics Committee (BREC) at the University of KwaZulu-Natal (UKZN), South Africa and the Committee on Human Research, Publication and Ethics (CHRPE) at the Kwame Nkrumah University of Science and Technology (KNUST) and KATH. The hospital (KATH), gave approval for the study in accordance with their gate keeping protocols. Study participation was voluntary, and respondents were assured of the right to withdraw at any point of the study with no consequences. Written consent was obtained from each respondent prior to data collection. Also, interviews were recorded with respondent’s permission. Interviewees were assigned codes (P 001-P0 eleven (11) to ensure anonymity. All the respondents were guaranteed confidentiality as well as anonymity during the publications of aspects of the study.

## Results

### Participant’s description

The women included in the study were eleven (11); aged 32 years to above 70 years. Three of the respondents were divorced, one was widowed, two were single and the rest were married. From their stories, time intervals for women to seek healthcare or consult the opinions of social networks were one (1) day (2), within 3–8 months (5), within 12–15 months (2), 24 months (1), and over 24 months (1). The other socio-demographics and clinical data of the respondents such as but not limited to religion, educational background, parity, family history of breast cancer, occupation, nature of symptom experienced, as well as stage of disease, are shown in S1 Table.

Women in this study shared their experiences with recognizing and appraising a BC symptom as a reason for late presentation. The themes that emerged were: symptom experience, knowledge of breast cancer, role of social life and networks, coping with a breast symptom and lastly intent to seek health care. The five main themes that emerged had sub-themes as shown in Table 1.

**Table 1:** Themes and sub-themes generated.

Themes	Sub-themes
Symptom experience	• Symptom discovery and nature • Reactions to the detected symptom • Interpretation of symptom
Knowledge of breast cancer	• Misconception and belief of breast cancer as a disease • Lack of knowledge about breast cancer and its outcome • Source of Knowledge
Role of social life and networks	• Disclosure and consultation of significant others about breast symptom • Priority to family responsibility and work
Coping with a breast symptom	• Denial • Optimistic bias about breast symptom • Religiosity
Intent to seek health care	• Prompts to seek help for a breast symptom • Pluralistic consultations

### Symptom experience

In this study, most women with ABC self–discovered their breast cancer symptoms and shared their journey from discovery to the label of a breast symptom. Few women also had their symptom discovered by a nurse during mass screening. The sub-themes emerged were: symptom discovery and nature, reactions to the detected symptom and interpretation of symptom.

#### Symptom discovery and nature

All the women in this study had a vivid imprint of the exact period they discovered the symptom as well as the nature of the symptom. Bathing time, grooming to work, and touching of the breast unconsciously were mentioned by women. Uniquely in this study, none of the eleven (11) women interviewed discovered her breast symptom through structured or intentional breast self-examination. Of the eleven (11) women, only three had initial vague symptoms such as nipple inversion, bloody nipple discharge, heaviness of the breast, painful breast (no lump) and intermittent breast itch. Pain was seen in only one woman out of the eight (8) women with initial breast lump and the rest (7) had painless lumps. Axillary lymphadenopathy was also noted by one woman with a painless lump. This is evident in the quote of Esi who felt heaviness in her right breast whiles grooming; she expressed:

*“I noticed the change on a Tuesday morning*, *just after Easter Monday*, *as I was grooming to go to the market; I felt that my right breast has become heavy and obviously bigger than the left one*. *Looking in the mirror*, *I was not wrong*. *Hence*, *I touched it and felt a lump like the size of ‘adwe’………*.*” (adwe in “Twi” means palm nut)*

Another woman also related:

*“Every month*, *I examine my breast*, *so I noticed the change very early*. *It was one evening while examining my breast in the bathroom; I felt heaviness and pain in my breast*. *I squeezed my nipple and a bloody discharge appeared*. *No lump though*. *Hence*, *I showed it to my husband; he also checked and confirmed the discharge” (Akua)*.

Aso who had her lump discovered by a nurse at a community mass screening had this to share:

*“One day*, *there was an announcement of a breast screening on 6*^*th*^ *March at …*. *[Name of town]*, *and since I lived near*, *I decided to attend*. *The nurse who examined me saw the lump in my breast……*.*”*

Seventy-five-year-old Dela is a retired teacher and a widow; her left breast nipple is naturally inverted since she was an adolescent. She noticed a vague symptom and she narrated:

*“This breast nipple (pointing to her left breast) is naturally inverted*. *It has been like that since my adolescence but suddenly*, *I felt persistent pain in my right breast*. *I felt both breast but there was no lump ……”*

#### Reactions to the detected symptom

Women in this study described their thoughts and feelings at the initial recognition of a breast symptom. Reactions described ranged from being scared and alarmed, anxious, worried to panic. This is evident in the following quotes:

*“*, *In fact*, *it scared me and got me panicked when I noticed the lump*, *I thought of it the whole day………” (Akoma)*.

*“I became anxious and alarmed when I noticed a bloody discharge staining my brassier…………” (Maame)*.

It was realized that extreme alarm, panic and anxious reactions influenced women to take action within few days of initial symptom discovery. For instance, Maame consulted a private practitioner the next day of symptom discovery. She had this to say:

*“I did not know what this was*. *I had a sleepless night and thought of it the whole night*. *First thing the following day*, *I reported quickly at the woman’s place (private Oncology practitioner) to get it resolved……”*

Other women with painless lumps also described their feeling as normal when they first noticed the bodily change. This is evident in Esi’s narration:

*“I was calm and normal with the initially lump*. *It was painless*, *and the breast looked normal*.*”*

From the above shared experiences, it appeared that the nature of bodily changes observed by women could be linked to their thoughts and feelings. Reactions such as worry, and scare influenced the women to follow up, thereby shortening delay. However, lack of worry prolonged appraisal delay. For instance, Emefa had this to say;

*“The lump was painless*, *and my breast looked normal*, *hence*, *I ignored it because*, *I was not worried*. *I did not consider it as something serious”*

#### Interpretation of symptom

Most women labeled painless breast lump as not serious and dangerous, normal or not a disease. In some women too, heaviness of the breast was attributed to hormonal changes such as ovulation period or sign of pregnancy. In the instance of Esi, she said:

*“I initially associated the heaviness to my menstruation as it was due. Again, the absence of the lump pain made me consider it as not serious. It should have been painful if it was anything abnormal or a disease”*.

Thirty-two years Ama also expressed:

*“I felt heaviness as well as painless lump in the breast*. *However*, *I had missed my menstruation for over one week; hence I considered the possibility of being pregnant*. *I was just waiting to be sure………* …*”*

In another instance, symptoms were labeled quickly as a cardinal sign of breast cancer or common illnesses such as malaria, leading to immediate follow up. Thus, recognition and poor-recognition of breast symptom contributed to either shortened or prolonged delay. On the contrary, correct recognition of a breast cancer symptom appeared to influence prompt entry to a health facility. This is illustrated in the following quotes:

*“In fact*, *I thought of possible cancer when I felt the lump; hence*, *I took it to the hospital immediately*. *I did not delay at all” (Akoma)*.

However, attribution of a recognized bodily change to a systemic illness led to delay as shown below:

“*As I narrated earlier*, *I had a bitter taste in my mouth*. *At that time too*, *there was pain in the breast*, *I deemed it as more of malaria*, *so I paid no attention to it” (Della)*.

### Knowledge of breast cancer

Awareness and knowledge about breast cancer as a disease and its outcome were explored among women. The experiences shared by the women in the study revealed a correlation between knowledge and symptom appraisal and help seeking. Misconception and belief of breast cancer as a disease; lack of knowledge about breast cancer and its outcome, and lastly, source of knowledge were the sub-themes identified.

#### Misconception and belief of breast cancer as a disease

Misconception and inaccurate knowledge and belief about breast cancer were expressed by some of the women in the study. Causes of breast cancer were strongly attributed to evil spirits, immoral lifestyle choices, and a bite of the breast. Emefa presumed that cancer results from God’s punishment and spiritual attacks because of specific actions such as extra marital immoral life. She narrated:

*“My belief is that cancer and other deadly diseases come as God’s punishment for leading an immoral life*. *I have been an honest and upright person since I got married about ten (10) years ago*, *so I have no reason to consider such impositions of spiritual diseases on my life*. *So*, *I had no cause to believe it was breast cancer*. *Therefore*, *I paid no attention for the lump for months” (Emefa)*.

In the case of Aso, she believed the cause of the disease as an attack by a family member through supernatural power intended to destroy or kill her. In turn, she sought deliverance and protection from a prayer camp.

“*I had a prophecy at church some months ago that a family witch (evil spirit) has bought breast cancer for me; just to destroy and kill me*. *I got delivered spiritually though*. *Hence*, *when it manifested physically as a lump in the breast*, *I took the spiritual route; I went for prayers*. *I deemed it as not a hospital disease; I ignored hospitals for more than a year” (Aso)*.

*“One of the issues was that I fought with my man (boyfriend) and he bit my breast*. *There was a cut which I dressed*. *Two weeks later*, *I found the lump*. *It was painful; but I linked it to the bite*. *Hence*, *I kept it to myself and took no action” (Amina)*.

In the interview, some women strongly believed that family history of breast cancer should be present for a woman to develop the disease. Also, those with a positive family history further linked the development of the disease to spiritual attacks or sharing personal items with the diseased person. Due to these erroneous beliefs, delayed presentation occurred. For instance, Dela said:

*“Since there is no history of breast cancer in my family*, *I didn’t really pay any particular attention to the cancer symptom*. *I knew once no one had it in my family*, *I should not also get it”*

*Amina also related*:

*“I deemed it more of a spiritual attack*. *My two sisters who directly come after me died with breast cancer*. *It was then my turn*. *Why was it lining us up*? *My eyes were opened at church of a spiritual link*, *hence*, *I moved from home to a prayer camp*. *I thought of fighting back spiritually……*.*”*

*Esi also narrated*:

*“I have a family history of cancer*, *but I shared nothing personal with those relatives*. *In fact*, *I never stayed with my father when he was diagnosed (prostate cancer) until he died*. *Hence*, *I never thought of the possibility of getting the disease in my life”*

It appeared that women’s negative view about breast cancer and its treatment prolonged presentation. Breast cancer was tagged by most women as incurable, deadly and a stigmatized disease. Also, those with prior negative experiences with the disease, causing suffering and death in known people had strong fatalistic views. The women’s perception of breast cancer led to avoidance. Consequently, women delayed seeking modern medicine to avoid breast cancer confirmation. This is illuminated in the quotes below:

*“I was afraid to be told it was breast cancer*, *a bad and incurable disease*. *Hence*, *I had no courage to start the hospital process” (Abena)*.

*“All I knew was that it was a dangerous disease and will lead to your death if you get it*. *I know three women who died exactly three (3) years after breast cancer was diagnosed*. *They really suffered*, *wound*, *pain……*. *Due to the associated suffering and death*, *I got scared and took my mind off it*. *What was the point going to the hospital*?*” (Dela)*.

#### Lack of knowledge about breast cancer and its outcome

In the interview, it was realized that some women seemed less receptive towards information about breast cancer before diagnosis. They had no knowledge of the cause, treatment and outcome of breast cancer. Also, information about breast examination and cardinal symptoms of the disease such as breast lump had not reached the women. This led to poor-recognition of symptoms which led to delayed presentation. For instance, Ewura said:

*“I had absolutely no idea what breast cancer was or any knowledge about the disease and symptoms before my diagnoses*. *I was totally ignorant about what was going on in my breast*. *Therefore*, *I did not take any action earlier”*

*“Mmmmmm*, *I had heard a little about cancer*, *but I had no idea what it was*. *Because I didn’t have any specific knowledge*, *my mind was not on the disease to give it a thought or attention*. *I didn’t know what was going on in my breast” (Serwaa)*.

Due to lack of specific knowledge about the disease, some women had no idea of the steps to take and this delayed their presentation. This is shown in the quote below:

*“I did not know what it was*, *the steps to take or the right place to go when I noticed the change in the breast; hence*, *it ends up badly……………” (Emefa)*.

Lack of personal experience of breast cancer was noted in some women; hence, the slow protracted course of breast cancer and its impact on women’s quality of life at the advanced stage was not known. Following diagnosis, most women regretted not attending to the symptom earlier.

“*Maybe if I had had much knowledge about breast cancer and its severity*, *I may have acted much quicker and reported the painless lump to the hospital*. *I regret for coming this late*. *Things are not easy [tearing]……” (Emefa)*.

*“I would have checked my breast regularly and taken the necessary steps to avoid this suffering had I known” (Ewura)*.

Most women were ignorant about the cause of the disease and expressed interest to know. This is evident in the quotes below:

*“I asked at the clinic about the possible cause of the disease or if it is associated with any food we eat or what*? *What really is the cause of BC*?*” (Esi)*.

#### Source of knowledge

Women shared their source of information as media (radio and television), church, health professional, and people around them. This is evident in Akoma’s story; she narrated:

*“I heard of BC through an awareness campaign on the media (radio and television) before my diagnosis*. *The message was centered on signs of possible BC such as a lump in any part of the breast*. *I saw the reality of the disease on TV”*

Maame also shared:

“There was an outreach program in church by some health workers a long time ago which informed my decision to check my breasts regularly”

Serwaa also expressed:

“*My daughter talked to me about the disease*. *There was a health talk on breast cancer at her school*. *Hence*, *she shared the information with me”*

### Role of social life and network

From the expressions of the women, opinions of significant others were sought to understand the noticed bodily changes. Women used social network interactions to make meaning of the situation and manage it. This influenced their time of presentation. Further, women social responsibilities such as family obligations and work also seemed to be an impeding factor. Two sub-themes emerged were disclosure and consultation of significant others about breast symptom and priority to family responsibility and work.

#### Disclosure and consultation of significant others about breast symptom

To understand the breast abnormality, women disclosed their symptoms to others. However, during disclosure, women considered only trusted individuals mainly for social and spiritual support while maintaining secrecy to avoid social consequences such as gossip, stigma, rejections, bad comments and spread of news. Selected social networks included first degree relatives (sister, mother, and daughter), spouse, friend, pastor, work owner and colleagues, and health worker mainly living in women’s community. In the case of Maame, symptom disclosure helped her to confirm her recognition and led her to seek health care:

*“To avoid gossips*, *I kept mulling over the issue for about four (4) months; the thought of discussing it with someone else was eating me up but I finally decided to talk to a trusted doctor living around*. *He looked and promptly decided there were enough differences between the two breasts to warrant a visit to the Breast Care Clinic of KATH”*.

*Abena also related*:

*“When I saw the lump*, *I was scared which led me to talk to my pastor*. *But my pastor assured me that it was not that dangerous*. *His wife had breast cancer and had mastectomy; it is not that scary*, *so I should take it to the hospital*. *So*, *I took the necessary steps”*.

*Amina shared*:

*“I had to tell my proprietor and colleagues at work*. *They asked me to report to the hospital and even offered to support me financial should the need be but……*. *hmmmm”*

Although seeking peoples’ opinions influenced women to seek early care, it is worth nothing that some women were directed to seek alternative therapies causing additional delays. This is evident in the quote below:

*“I later showed the lump to my best friend and asked her to keep it a secret*. *She directed me to a herbalist and I spent about 6 months there but my breast got worse with a wound and a swollen arm” (Amina)*

Two women paid no attention to their lumps for over 12 months because they were informed that it was normal for every woman to have a lump in the breast. Hence, breast cancer symptoms were misinterpreted as normal developmental changes. For instance, Ewura shared:

*“I phoned my mother to inform her about the lump*, *but she assured me that it was a normal thing*. *Every woman has a lump in her breast*. *I had no cause to worry*. *I did nothing about it”*

Due to Ghana’s health care system, women are expected to follow referral channels to a tertiary facility. Hence, most of the women advised by their social network to seek modern medicine sought care at health centers, district and regional hospitals as well as a private oncology facility. However, some women experienced delays due to provider’s poor recognition of breast cancer symptom, perceived misdiagnosis and mismanagement. This is illustrated in the quotes below:

*“For me*, *I started early but the path I took delayed me*. *I did not start with a private clinic*, *but a government hospital staffed with qualified doctors*. *Right from the district to the regional hospitals*, *the doctors could not recognize breast cancer with all the signs I presented*. *I was told everything was normal in the breast” (Akoma)*.

*I visited a regional hospital*. *The doctor did a physical examination and then some tests*. *After studying the results*, *he did a surgery to remove the lump and discharged me*. *After a year*, *the disease recurred terribly; with pain and a festering wound” (Ewura)*.

#### Priority to family responsibility and work

Women’s social responsibilities regarding commitments to work, caring for children and funeral celebrations of close relatives and their impact on delay presentation were also noted in the women’s stories. Due to the above social obligations, some women postponed going to the hospital for months. In the case of Amina, commitments to family (children’s welfare and funerals of two close relatives) were apparent, leading her to delay presentation irrespective of advice from work to seek care as well as her personal experience with breast cancer and its outcome. She shared:

*“hmmmm…*., *the whole thing was complicated madam*. *Thou I had lost my two sisters to breast cancer; I ignored this important issue as if I never heard or experienced it*. *I was as well offered advice and help at work……*. *but hmmm……*. *I also concentrated all my efforts on taking care of my children”*

She continued to say:

*“At that same period*, *a lot of incidents were going on in my house; the father of my daughter died; my sister who came right after me also died of breast cancer*. *We had to prepare for all these funerals*. *The pain started but I ignored it [weeping]…”*

Ewura also expressed:

*My problem stems from my work schedule; I leave home at 5am and sometimes get home as late as 1 am*. *Due to this*, *I would be totally exhausted most days by the time I get home*. *If I tell you I had been paying attention to the lump seriously*, *I would be misleading you*.

### Coping with a breast symptom

This theme describes how maladaptive coping strategies employed by women prolonged presentation of initial breast symptom. The sub-themes identified were: denial, optimistic bias and religiosity.

#### Denial

Denial was a coping mechanism that influenced women symptom appraisal. Perception of breast cancer as an incurable and fatal disease led to denial of accepting the possibility of cancer. This is evident in the quotes below:

*“I did not believe it (lump) could be cancer*. *Why me*? *It cannot be*. *I just shut it off my mind*. *I did not want to go to the doctor to be told I have cancer*. *I lived my normal life as if all was well” (Aso)*.

*“I never thought of a possibility of breast cancer*. *Not me*. *Cancer diseases are deadly*, *and they start seriously and differently*. *Not painless as this*. *I moved on with my life just like before*: *(Emefa)*.

#### Optimistic bias about breast symptom

In the study, women employed past experiences with breast problems to interpret the recognized symptoms. Thus, optimistic bias towards the symptom was noted. For instance, Della shared:

“*I experienced breast problem whenever I gave birth with breast feeding*, *I deemed it as more of the same issue*. *Hence*, *I took it less serious and left it”*

*“Due to my previous experiences*, *I did not take the heaviness of the breast serious*. *My breast heaviness has been cyclical*. *I therefore took it as one of those things” (Esi)*.

#### Religiosity

All the women in the study were Christians and the impact of a person’s religious belief on delayed presentation was shown in the study. In some instances, reassurance messages from women’s social network were religiously related. Hence, some women did not follow up based on the assured messages they received from others that God has the supernatural power to miraculously melt a breast lump. This is illustrated in the quotes below:

*“I know God takes care of our disease*. *This was my belief*. *I had that faith; I therefore committed everything to prayer and fasting*. *My faith kept me going ……*.*” (Della)*.

*“My sister assured me of God’s miracle*. *Hence*, *I waited on God as a Christian*. *I went for prayer sections and applied anointing oils*. *I became so much relaxed just believing in God’s miracles” (Amina)*.

### Intent to seek health care

This theme describes women’s decision towards care seeking and factors that influenced the women attention to the symptom. Prompts to seeking help for a breast symptom and pluralistic consultation emerged from the data.

#### Prompts to seeking help for a breast symptom

A few women followed-up for immediate medical attention on discovery of bodily changes. However, most women unconsciously monitored the initial symptoms for additional symptoms. Worsening of initial symptoms signaled or prompted the women to act. Emefa shared her experience:

*“I was unconsciously monitoring; the skin of the breast changed to an orange*, *it was getting worse; I asked myself*, *what is this*? *I had to follow up” (Esi)*.

Another woman also related:

*“Things were getting bad and that triggered me to seek health care” (Emefa)*.

#### Pluralistic consultations

Due to the pluralistic nature of the Ghanaian health care system, women had access and the choice for complementary and alternative medicine as well as private health facilities. Hence, herbal medicine, private consultations, spiritual care and over the counter drugs from pharmaceutical shops were used in lieu of modern medicine. This caused delay in various stages of the disease such as pre-diagnosis, intra-diagnosis and post-diagnosis. Choice of therapy depended on women’s prior personal experience, recommendations from trusted people and influence from the media. Adoption of alternative medicine was also influenced by lack of confidence in modern health care systems because of perceived mismanagement. These are illustrated in the quotes below:

*“I heard an announcement on the radio regarding the woman’s place (Private Oncology Clinic)*. *Hence*, *I went there to seek care for the lump*. *This is the popular place we all know*. *She did a mammogram and only removed the lump*, *but I was not informed that I have breast cancer*. *I was reviewed post-surgery for more than 12 months*, *spent so much money and got discharged*. *However*, *the problem recurred in a worse form*. *Now*, *they say that the disease has gone to my lungs; thus*, *my disease has advanced” (Serwaa)*.

*“I first reported the pain to a drug store (pharmacy) in my area; I was given some pain killers for some time*. *Then*, *I reported at KATH*, *but the doctors went on strike in the process*. *Hence*, *a co-tenant introduced me to a herbalist who successfully treated her with a breast problem at …*. *[Name of town*.*]*. *I went there*, *and I was put on herbal concoction for three (3) months but nothing good came out” (Aso)*.

*“I started at a prayer camp*. *I was given anointing oil to smear on the breast and some spiritual directions to follow to get the lump out of the breast*, *but things did not go well*. *Later*, *someone directed me to buy some of these foreign supplements*. *I used it for 2 months*, *but things got bad” (Amina)*.

## Discussion

The study explored and described the recognition and appraisal of BC symptom as a reason for delayed presentation among women diagnosed with ABC. Findings from this study highlight the significance of symptom appraisal as seen in previous studies [14, 23]. Importantly, essential facilitators necessary for symptom appraisal within the socio-cultural context of Ghana has been illuminated. These include the discovery and recognition of the distinct symptom as abnormal, relating the symptom directly to the breast, inferring illness and the perceived intent of help seeking. Notably, the eleven (11) women in our study had overlapping reason for appraisal and time interval; however, symptom appraisal among the women occurred in two main stages, thus, first subjective understanding and later interactive (objective) understanding of the symptom based on cognitive and psycho-socio-cultural characteristics. Apparently, symptom labeling among women was poor not only in women with vague symptoms as shown in earlier studies [12, 23] but unique in this study, seven (7) out of eight (8) women with an initial symptom of a lump also experiences time interval.

Previous authors have established a correlation between low awareness of BC and time point interval [12, 14, 17, 32]. Although majority of women in our study were aware of breast cancer, it appeared that knowledge deficit as well as misconceptions and beliefs about the disease was high leading women to experience appraisal interval in interpretation of the symptom as abnormal. As confirmed by previous studies, Ghanaian women knowledge, beliefs and attitudes about BC is generally poor [33, 34]; this may reflect the reason for poor symptom appraisal in this study. Consistent with prior studies, breast lump was a common symptom in our study [14, 17]; but it was not perceived as serious or abnormal until sensory and other physical signs such as pain, skin changes, wound, lymphoedema and increase in size of lump were experienced. Evidently, pain from breast lumps is known to stimulate women’s help-seeking habit [14, 15, 17, 32]; congruent with our findings, the presence of pain led some women to interpret the lump as serious, provoking women to take action. Although the presence of pain was a triggering factor for most women in our study, prompting them to seek help, one woman ignored the pain and experienced time interval of 12 months. Our finding highlights the need for effective awareness interventions to broaden women’s knowledge on both cardinal and vague symptoms of BC to promote early detection.

Some women in our study unconsciously monitored the progress of the symptom before deciding to seek help. Social obligations such as priority to work and family responsibilities such as mothering and funerals were common impeding factors that influenced the decision to seek care. Evidently, women are known to prioritize other matters above their own health [35, 36], however, in the instance of BC, limited evidence is available on how work, parenting and family’s social functions may influence health seeking behavior among women [17, 37]. Uniquely in our study, employer and working colleagues initiated a significant role to shorten time interval after disclosure. For at least, one woman in our study was offered advice and financial support to seek care by her proprietor and co-workers, however, she still experienced time interval and did not seek care due to family issues, specifically, mothering and a funeral. Research exploring the role of parenting and social functions in appraisal and time interval within the context of BC is required to comprehensively understand the phenomenon.

Perceived limited risk of developing BC in the absence of positive family history was another factor noted in our study and others [17, 23], contributing to poor symptom recognition and prolonged time interval. However, in the presence of positive family history, the Ghanaian women still perceived limited risk of developing the disease due to misconceptions and beliefs such as perceiving BC as communicable as well as linking the disease to spiritual influence. In contrast to our findings, the presence of positive family history of BC increased women awareness of their increase susceptibility to BC [38]. This likely affected their early detection practices which in turn influenced early detection among women in previous studies [14]. The absence of genetic counseling and follow-up post diagnosis of BC within a family in Ghana [39, 40] could contribute to the erroneous knowledge and beliefs of the disease held by women in our study. Although genetic counseling and testing is not available in Ghana, immediate intervention to make patient’s family knowledgeable about BC and its early detection practices following diagnosis is required. This will promote early detection of BC among women.

In the literature, definition of delay as theorized is not only centered on the patient [20]. Total patient delay has been described by Andersen et al [20] in five (5) stages to include physician or system delay. In our study, poor symptom appraisal among women was not the only facilitator of time interval; perceived poor symptom recognition, misdiagnosis and mismanagement by both government and private health care practitioners as well as community pharmacists also led to diagnosis interval as seen in other studies [17, 41]. Uniquely in our study, community pharmacists played a key role to appraisal and time interval. For at least one woman was managed at a community pharmacy on analgesic for weeks after disclosure of a breast pain; leading to appraisal and time interval with a poor outcome. This emphasized that early detection of BC symptom at all levels of health care is paramount for diagnosis, and management. The current findings show that health care provider’s knowledge on BC and their symptom appraisal are very significant in diagnosis of women presenting with a breast symptom. This study suggests the need to educate the public and health care providers on BC to reduce the likelihood of late diagnosis of the disease. Also, further research that explores the role of community pharmacists in breast symptom appraisal is required as this appears a key to early detection of BC. In addition, a future study to explore the role of health facilities in appraisal, time and diagnosis intervals of BC is needed as this seems significant to early detection of BC in Ghana.

In his theory of total patient’s delay [20], lived experience of the individual and others was noted as a core factor in symptom appraisal. As seen in this study, women’s own previous experiences with a breast related problem and knowing someone with BC lulled women into a false sense of hope [14], leading to denial of accepting the possibility of cancer. This was seen as a strategy employed by women to cope with the recognized bodily change [23]. The incidence of cancer in Ghana is relatively increasing and BC is now the top diagnosed cancer [4]; likely making a personal encounter with BC relatively common within the Ghanaian community. Due to the fatal outlook and poor outcome of BC which is stage and mastectomy related [2, 42], a social norm of doom is often perceived [14]. Hence, the unfavorable outcome and frequent death of women diagnosed with BC appeared as a constant reminder to women with new breast symptom, that the disease is incurable and untreatable [42, 43]. Therefore, to avoid cancer confirmation, women did not seek care following symptom recognition. As in other communities [14], fatalism also exists within the Ghanaian community as a sign of hopelessness [34]. Women in our study had a strong fatalistic view. This triggered fear as the women chose not to seek help to avoid malignant confirmation.

Consistent with other findings [12, 17], the worsening or additional breast symptoms experienced by women in our study elicited a sense of anxiousness, worry and panic and this stimulated women to consult their social network for advice. The process of making a decision is a component of appraisal [14] and studies that applied Andersen appraisal posited that bodily sensations are also influenced by the psycho-socio-cultural experiences of the individual [14, 15]. Culturally, the perceived stigma and rejection associated with BC within the Ghanaian society emerged in this study. This was a core reason why the Ghanaian woman in this study disclosed her symptom to a select few based-on trusts, basically for endorsement of body abnormality, social and spiritual support while maintaining secrecy. Our study affirms the findings of other studies on the significant role played by social networks in fostering timely presentation as well as endorsing ill health [17]. Disclosure to a family member, spouse and pastor was a significant trigger to seek health care in our study. Also, where validation of breast changes was made by a health care provider (midwife or medical doctor), women sought prompt entry to tertiary health care centers; hence, time and diagnosis intervals were shortened. Uniquely in our study, disclosure to friends triggered prompt help-seeking, however, women in the study were led to seek help from herbalist and prayer camps; hence appraisal and time interval resulted. In our study, secrecy was maintained in disclosure due to the perceived negative social consequences of BC diagnosis such as gossips, isolation and unfavorable comments from the public. Our findings thus highlight the limited education on BC at the community level and set the stage of BC awareness campaigns aiming at a larger audience from women to all members within a community with a message on cure and favorable prognosis associated with early diagnosis inclusive.

Another factor seen to contribute to time and diagnosis interval was the pluralistic nature of the Ghanaian health care system. The Ghanaian woman in this study had the choice for alternative therapies. Therefore, herbal medicine and prayer camps were frequently used in all stages of the disease including the pre-diagnosis stage. The type of therapy women accessed for initial symptom was influenced by the perceived care each provided, media, prior individual experience and social network recommendation. These factors led to a delay in entry to a health care facility as shown in other studies [15, 44]. Adoption of an alternative therapy was further influenced by lack of trust in modern medicine as possible initial misdiagnosis and mismanagement by public and private practitioners were reported in this study. Women in our study reported frequent use of herbalists and religious leaders, suggesting that such people could play a significant role in healthcare entry within the communities. Further research is required to examine the need for provider training in assessment of potential symptoms of BC. Further, misconceptions and beliefs led some women to access traditional medicine and this has been reported in cancer studies across African communities [43, 45, 46]. As reported in some Nigerian studies, religious belief and prayer also contributed to appraisal interval and led some women in our study to experience time interval [16, 23]. As noted by previous authors, religiosity is a significant part of the socio-cultural context of Ghana and this should be acknowledged by health professionals [27].

**Fig 1:**
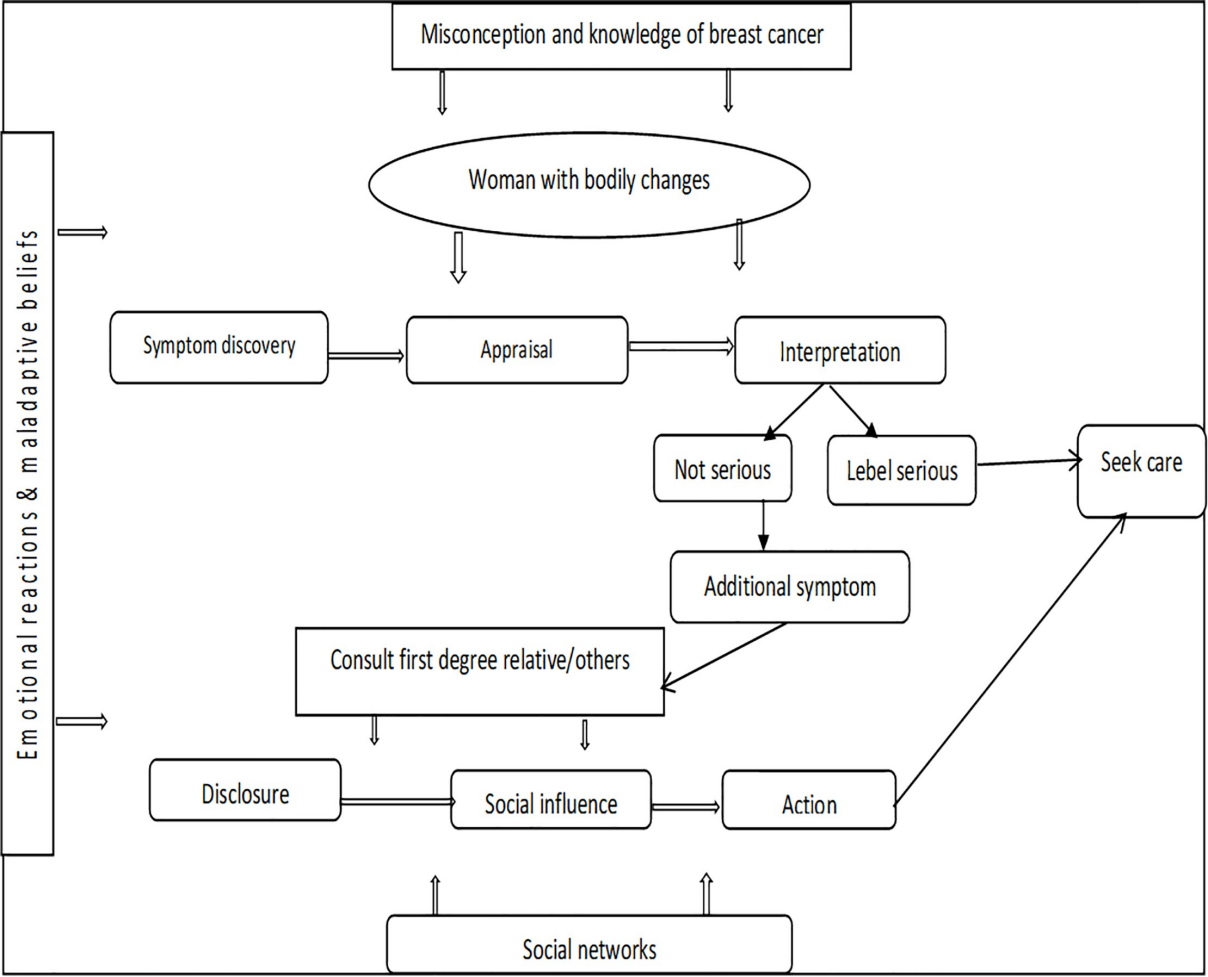
Cognitive and psycho-socio-cultural model of breast cancer symptom appraisal and recognition.

Evidence generated from this study provided a theoretical approach to explain BC symptom discovery and appraisal as shared by Ghanaian women. This led to the development of a symptom recognition and appraisal model as shown in Fig 1. This was based on previous models such as the Andersen Model of Total Patient Delay [20], the Model of Socio-Cultural Interpretation of Symptoms [47] and the Psycho-socio-cultural model of symptom appraisal [44]. Our proposed model is contextualized, adding cognitive and emotional factors as well as other significant constructs to existing models around symptom appraisal. Fig 1 shows the cognitive and psycho-socio-cultural model of breast cancer symptom recognition and appraisal. This illustrates from a woman with a bodily change to the discovery of the breast symptom, appraisal and interpretation of the symptom as serious or not serious as well as consultation and disclosure to significant others, validation, submission to social influence and actions towards care seeking for the symptom. This process is facilitated by knowledge of BC, misconceptions and maladaptive beliefs about the disease, emotional reactions towards the symptoms as well as impact of social networks. These occurred within the context of cognitive, emotional, cultural and social factors. The emotional context reflects how the Ghanaian women reacted to and coped with the initial bodily changes based on their own previous experiences with a breast problem, denial and faith.

Our findings suggest awareness campaigns to address misconceptions about BC. Our model would serve as a framework for designing such awareness interventions. Educational messages should be designed to include BC as a disease, symptoms, risk factors, treatment, possible cures and favorable prognosis associated with early detection. Projection of BC as a terminal incurable disease within the communities should be limited. Awareness programs should be extended to include health practitioners, religious leaders as well as alternative medicine practitioners due to the multiple levels of influence that affect early detection. To achieve early detection of BC, educational intervention for family members of patients following a diagnosis of BC within health care facilities is paramount. Also, effort must be tailored at screening services within the health care system to promote early detection of BC.

In summary, findings from the study indicate that recognition of BC symptom is relatively poor and remains a public health challenge in Ghana. Over-lapping factors such as misconceptions and maladaptive beliefs, lack of knowledge on BC as well as psycho-socio-cultural factors influence symptom recognition and appraisal. To have a better understanding of the phenomenon, a study to explore potential facilitators to earlier presentation among women diagnosed with early stage BC in Ghana is recommended.

## Limitations and strength

We acknowledge some limitations. We recognize that an appreciable number of Ghanaian women with ABC may never reach the palliative care clinic which was the recruitment outlet for the study due to the referral system. However, the study provides rich and in-depth information on women’s experience with symptom recognition and appraisal as well as key factors influencing appraisal and time intervals within the socio-cultural context of Ghana, strength of qualitative research. The findings can be used to improve early detection of BC in Ghana and other countries with similar context. Further, all the women were diagnosed a few months to years prior to being interviewed, hence, retrospective recall may affect accuracy of shared information. However, women were recruited at different periods of diagnosis and similar experiences from women with recent and past diagnosis were heard.

## Conclusion

Breast cancer patients in Ghana experienced long appraisal intervals from initial symptom onset, interpretation, labeling and initiating help-seeking. Due to poor symptoms recognition and appraisal, a time interval from three (3) months to over 24 months was observed in the study sample, leading to an advanced presentation. Misconceptions and maladaptive beliefs about BC influenced the choice of therapies in the study sample. For instance, fatalistic beliefs led women to access alternative therapies such as spiritual and traditional medicine for months, causing a time interval in hospital entry. This resulted in an advanced presentation. In addition, a lack of knowledge about BC caused women to ignore possible BC symptoms and this delayed their help-seeking process. Opinions of family members and friends led women to ignore symptoms or sought alternative therapies. However, a prompt hospital entry within a day of symptom discovery was observed in women who had seen BC before on television and those who consulted other people with a clinical background or personal experience with the disease. Knowledge deficiency among clinical and allied health professionals such as community pharmacists led to time interval, misdiagnosis and mismanagement. Hence, advanced stage presentation resulted. To achieve early detection of BC, educational and screening interventions should be provided for Ghanaian women and their significant relations such as family members and friends. Poor knowledge of BC and its symptom among health professionals should be acknowledged and addressed accordingly. Spiritual leaders and traditional herbalists are significant stakeholders in BC diagnosis in Ghana. They should, therefore be acknowledged when initiating educational interventions to improve early detection of BC in Ghana. Future studies exploring the roles of community pharmacists as well as health facilities in delayed presentation are required in Ghana.

## Supporting information

S1 Table Participants profile. (DOCX)

S1 File Interview guide English version. (DOCX)

S2 File Interview guide in original language. (DOCX)
