# Supplementary material for: Recognizing and appraising symptoms of breast cancer as a reason for delayed presentation in Ghanaian women: A qualitative study
Source: PLoS One. 2019 Jan 9;14(1):e0208773. doi: 10.1371/journal.pone.0208773 (PMC6326484; doi:10.1371/journal.pone.0208773)
Supplement: S1 Table — (DOCX) [file pone.0208773.s001.docx]

**S1 Table: Participant’s Profile and Clinical Features**

| **Pseudonyms** | **Age range**  **(years)** | **Educational background** | **Religion** | **Marital**  **Status** | **Number of Children** | **Occupation** | **Family history of breast cancer** | **Nature of initial breast symptom** | | **Time interval to presentation**  **(Months)** | **Pathology Report** | **Stage diagnosed** | **Number of months diagnosed** | **Status during data collection^*^** | **Current status^**^** |
| --- | --- | --- | --- | --- | --- | --- | --- | --- | --- | --- | --- | --- | --- | --- | --- |
|  |  |  |  |  |  |  |  | **Lump** |  |  |  |  |  |  |  |
| Esi | 41-50 | Secondary | Christian | Divorced | 3 | Trader | Yes | Painless | Heaviness | 4 | Malignant | III B | 5 | Well | Alive |
| Akoma | 51-60 | Middle | Christian | Married | 4 | Trader | No | Painless  &  Axillary lump |  | 7 | Malignant | III B | 6 | Well | Alive |
| Serwaa | 51-60 | Middle | Christian | Married | 6 | Unemployed | Yes | Painless |  | 8 | Malignant | IV | 36 | Well | Died |
| Akua | 41-50 | Middle | Christian | Married | 3 | Self employed | No | No lump | Heaviness, painful breast, & bloody nipple discharge | 1 day | Malignant | IV | 36 | Fairly ill | Died |
| Emefa | 41-50 | Secondary | Christian | Married | 1 | Trader | No | Painless |  | 24 | Malignant | IV | 4 | Fairly ill | Alive |
| Dela | ≥71 | Tertiary | Christian | Widowed | 7 | Retired teacher | No |  | Painful breast | 3 | Malignant | IV | 10 | Fairly ill | Died |
| Ewura | 41-50 | Middle | Christian | Single | 2 | Self employed | Yes | Painless |  | 12 | Malignant | IV | 19 | Well | Alive |
| Maame | 51-60 | Middle | Christian | Divorced | 1 | Unemployed | No | No lump | Bloody nipple discharge & inverted nipple | 1 day | Malignant | IV | 3 | Well | Alive |
| Amina | 51-60 | Secondary | Christian | Divorced | 3 | Unemployed | Yes | Painful |  | 24 | Malignant | IV | 36 | Fairly ill | Died |
| Aso | 41-50 | Middle | Christian | Married | 3 | Trader | No | Painless |  | 27 | Malignant | IV | 24 | Fairly ill | Died |
| Abena | 31-40 | Middle | Christian | Single | - | Trader | No | Painless | Heaviness | 3 | Malignant | III A | 2 | Well | Alive |

^*^January-May, 2018

^**^June, 2018
