# Supplementary material for: Recognizing and appraising symptoms of breast cancer as a reason for delayed presentation in Ghanaian women: A qualitative study
Source: PLoS One. 2019 Jan 9;14(1):e0208773. doi: 10.1371/journal.pone.0208773 (PMC6326484; doi:10.1371/journal.pone.0208773)
Supplement: S1 File — (DOCX) [file pone.0208773.s002.docx]

**Recognizing and appraising symptoms of breast cancer as a reason for delayed presentation in Ghanaian women: A qualitative study**

## Semi-structured interview guide.

## Introduction

Thank you for agreeing to share your experiential journey on how you discovered your breast symptoms and your decisions to seek help with me. Before we start our conversation, I want to assure you that your interview responses will be kept confidential and will not be shared with your health care team. Your responses will be combined with that of other study participants and will not be linked with your name or any identifiable information that can be used to trace you. You may skip any questions you don’t want to answer and you may end the interview at any time. You may also decide to withdraw from this study at any time without any consequences.

We will like to record this interview to make sure that we listen to your responses correctly. We will write down your responses verbatim or base on the meaning of your comments. We shall share the recording and the transcripts with a person who is good in Twi and English language to review your responses which we have written down. This will ensure that we have written your responses correctly. Your name will not be associated with the recording or on the transcript. We will assign you a number as your study code. If your name or any identifiable information comes on the transcript, we will delete it before sharing it with other individuals who may have access to the study data.

We will use false name to replace the number assigned to you when quoting your expressions in any part of the study or during publication of aspects of the study. We will keep all your recording and transcripts with a password and in a cabinet under lock and key for 5 years. All documents bearing any of your identifiable information will be kept separately; hence, no one can trace the documents to you. All the documents will be destroyed after the storage period. You will have to sign a consent form which I will witness showing your willingness to participate in the study.

Do I have your permission to start the interview?

[If No, thank participant for time and end the session] [If yes, continue with the interview]

Thank you, before I start the interview, pleases sign the consent form.

Thank you for consenting for participation. Before I start to record, I will ask some general questions about you.

**Section A**

1. Your age (Years)………………………….
2. Place of residence………………………………
3. Religion……………………………………………..
4. Highest level of education……………………………………….
5. Occupation…………………………………………………..
6. Marital status…………………………………………………..
7. Number of children…………………………………………
8. Family history of cancer………………………………………..
9. If yes, relation……………………………………………………...
10. Type of cancer……………………………………………
11. How long have been diagnosed of breast cancer……………………………
12. When were you referred to the palliative care clinic………………………

Thank you for sharing your background with me. Now, do I have your permission to start the recording?

[If No, thank participant for time and end the session] [If yes, continue with the interview]

Now that the recording has started, please say “Yes” to confirm that you approve of me recording the interview

**Section B**

**Main question: Please can you share with me your story before you were diagnosed with breast cancer?**

Probes

1. How did you discover your breast cancer symptom?
2. Screening? Why? When?
3. So what happened next?
4. Feeling when you noticed it?
5. Sought any help? When? Where? Who? Why?
6. Can you tell be about your journey from the discovery to diagnosis
7. Treatment before hospital? Why, Where? When?
8. Seeking medical care? Where? When? Why?
9. Why did you come late?
10. Did you inform anybody that you have a breast symptom?
11. Family member?
12. Spouse?
13. Friend?
14. Why? When?

Those are all the questions I have for you today. Is there anything else you would like to share with the research team about breast cancer screening or this study?
