# Supplementary material for: Recognizing and appraising symptoms of breast cancer as a reason for delayed presentation in Ghanaian women: A qualitative study
Source: PLoS One. 2019 Jan 9;14(1):e0208773. doi: 10.1371/journal.pone.0208773 (PMC6326484; doi:10.1371/journal.pone.0208773)
Supplement: S2 File — (DOCX) [file pone.0208773.s003.docx]

**Recognizing and appraising symptoms of breast cancer as a reason for delayed presentation in Ghanaian women: A qualitative study**

## Semi-structured interview guide-Twi Version.

**Nniamnsɛm**

Yɛda wase sɛ wagye atom sɛ wobɛka sɛnea wohunu sɛ sɛnkyerɛne bi da adi wɔ wonufu mu ne nea ɛyeɛ a ɛma wo sii gyinayɛ sɛ wobɛhwehwɛ mmoa. Ansa na yɛbɛ firi yɛn nkɔmmɔbɔ no ase no, mepɛ sɛ mema wohunu sɛ mmue biara a wode bɛma no mede bɛsie a obiara nnya ho kwan nte bi, afei menka ho asɛm biara nkyerɛ ayaresafo kuw a wɔhwɛ wo no. Yɛde wo mmueɛ ne afoforɔ a yɛne wɔn bɛtwetwe nkɔmmɔ no mmueɛ bɛbom na yɛnfa wo din anaa bribi foforɔ a ɛbɛma yahunu wakyi kwan nka nsɛm a yɛbɛtwerɛ no ho. Wowɔ hokwan sɛ wogyae asɛm bisa biara wonpɛ sɛ woma ho mmueɛ na wobɛtumi agyae nkɔmmɔ twetweɛ no bere biara a wopɛ. Wobɛtumi asi gyinae sɛ wonfa wo nhyɛ nhwehwɛ mu yi mu bio bere biara, a obiara ntumi nyɛ wo hwee.

Yɛpɛ sɛ yɛtwe nkɔmmɔbɔ no gu afiri so na yatumi atie wo mmuae no yie. Yɛbɛ twerɛ wommuae no pɛpɛɛpɛ anaa sɛ yɛbɛ gyina nteaseɛ a ɛwom so. Yɛde wo mmuae no bɛma obi a wɔnim Brofo ne Twi kasa wama wahwɛ wo mmuae a yatwerɛ ato hɔ no. Yie bɛma yahunu sɛ yatwerɛ wo mmuae no pɛpɛɛpɛ. Yɛnfa wo din nhyɛ nsɛm a yatwe agu afiri so anaa nsano-ntwereɛ no so. Yɛde nuba bɛyɛ agyinaeyɛhyɛdeɛ ama wo. Sɛ ɛkɔba sɛ wo din anaa nnɛama foforo a ɛfa wo ho pue wɔ nsano-ntwerɛ no so a, yɛbɛ popa ansa na obi foforo nsa aka nsɛm a yɛde reyɛ nhwehwɛ mu no.

Yɛde ɛdin a wasesa no bɛsi nuba no a yɛde yɛɛ wagyinayɛhyɛdeɛ no anan mu bere a yɛre twe adwene asi wanum asɛm so wɔ adesua anaa nhwehwɛm yi fa biara so, saa ara na yɛbɛyɛ bere a yɛde adesusa no ɛto dwa anaa yɛrema amansan nyinaa a kan bi. Yɛde ‘password’ bɛto wanum nsɛm a watwe anaa nsano-ntwerɛ no so de asei mfei 5. Wɔbɛkora wo ho nsem foforɔ a watwerɛ a to hɔ no so wɔ beaɛ soronko a obiara ntumi nhunu wakyi kwan. sɛ berɛ wahyɛ no so a, wɔbɛseɛ nsɛm ahodoɔ a ɛfa wo ho nyinaa. Wubɛtim anaa wobɛ san wɔ krataa yi so de akyerɛ me sɛ wode wo ho bɛhyɛ nhwɛhwɛmu.

Me srɛ wo mɛtumi afiri nkɔmmɔbɔ yi ase?

[Sɛ ɔka daabi a, da no ase na twa nkɔmmɔbɔ no so] [sɛ ɔka aane a toa nkɔmmɔbɔ no so]

Meda wase, ansa na mefiri nkɔmmɔbɔ no ase no, san krataa a ɛkyerɛ sɛ wagye a tom no so.

Meda wase sɛ wagye a tom sɛ wode wo ho bɛhyɛ dwumadie no mu, ansa na mɛkyere nsɛm yi agu afiri so no, mɛbusa wo ho nsem kakera.

**ɔfa A**

1. Mfei a wadi (mfe) .................................................................
2. Baabi a wote .........................................................................
3. ɔsom ...................................................................................
4. baabi a wakɔ sukuu akɔ du...........................................................
5. Adwuma ......................................................................................
6. Awareɛ mu gyinabea ....................................................................
7. Mma dodoɔ ...................................................................................
8. Abusua abakɔsem wɔ koram yareɛ ho ...........................................
9. Sɛ aane a, ɔyɛ wo dɛn? ......................................................
10. Kokoram yareɛ no mu deɛ ɛwɔ hen? ...................................
11. Wɔdaa no adi kyerɛ wo sɛ wowɔ nufoɔ mu kokoram mfei sɛn ni? .........................
12. Berɛ bɛn na wɔkyerɛ wo sɛ kɔ ayaresabea? ..............................................

Meda wase sɛ wama mahunu woho nsɛm, afei, mɛtumi afiri aseɛ akyere nkɔmmɔbɔ no agu afiri so?

[Sɛ ɔka daabi a, da no ase na twa nkɔmmɔbɔ no so] [sɛ ɔka aane a toa nkɔmmɔbɔ no so]

Afei a mafiri aseɛ ɛkyere nsem yi gu afiri yi so no, ka sɛ “Aane” fa kyerɛ sɛ wogye tom.

**Ɔfa B**

**Asɛmbisa titiriw: mepakyew wubɛtumi a aka w’abrabɔ mu nsɛm kakera akyerɛ me ansa na ɔka akyerɛ wo sɛ wanya nufoɔ mu kokoram?**

**Nhwehwɛmu**

1. Nsɛnkyerɛne bɛn na ɛdaa adi sɛ wowɔ nufoɔ mu kokoram?
2. Nhwehwɛmu? Adɛn? Berɛ ben?
3. Dɛn na ɛsii bio?
4. Berɛ bɛn na wotee nka?
5. Wohwehwɛɛ mmoa bi? Berɛ ben? ɛhefa? Hwan? Adɛn?
6. Wubɛtumi aka deɛ ɛsii berɛ a wuhunui ne berɛ a Dokta sii so dua?
7. Ayaresa ansa na wokɔ ayaresabea? Adɛn? Ɛhefa? Berɛ ben?
8. Wo hwehwɛ ayaresa? ɛhefa? Berɛ ben? Adɛn?
9. Adɛn nti na wokyɛɛ ansa na woba?
10. woka kyerɛ obi sɛ sɛnkyerɛne bi da adi wɔ wo nufu mu?
11. obusuani?
12. Wo hokafo?
13. Wa damfo?
14. Adɛn? ɛhefa?

Wie ne nsɛmmisa a mepɛ sɛ mebisa wo nnɛ. Biribi foforɔ bi wɔ hɔ a ɛfa nufuo mu kokoram ho a wopɛ sɛ woka kyerɛ nhwehwɛmu kuo yi wɔ adesua wie mu?

**Medaasa**
